# Tuberculin PPD Potency Assays in Naturally Infected Tuberculous Cattle as a Quality Control Measure in the Irish Bovine Tuberculosis Eradication Programme

**DOI:** 10.3389/fvets.2019.00328

**Published:** 2019-10-01

**Authors:** Anthony Duignan, Kevin Kenny, Douwe Bakker, Margaret Good

**Affiliations:** ^1^Department of Agriculture, Food and the Marine, Dublin, Ireland; ^2^Central Veterinary Research Laboratory, Department of Agriculture, Food and the Marine, Celbridge, Ireland; ^3^Independent Researcher and Private Consultant, Lelystad, Netherlands; ^4^Independent Researcher and Private Consultant (Retired From Department of Agriculture, Food and the Marine), Dún Laoghaire, Ireland

**Keywords:** quality control, tuberculin, PPD, tuberculosis, bovine, potency, Ireland

## Abstract

The Irish Bovine Tuberculosis (bTB) eradication programme operates under national legislation and fulfills OIE and EU trade requirements. Tuberculin purified protein derivative (PPD), a preparation obtained from the heat-treated products of growth and lysis of *Mycobacterium bovis* or *Mycobacterium avium* (as appropriate), is critical to the diagnosis of tuberculosis (TB). Standardization of Tuberculin PPD potency, the relative activity in sensitized animals compared to a reference standard, is essential to underpin the reliability of certification for international trade and to ensure that disease eradication programmes are effective and efficient. A Bovine International Standard Tuberculin PPD (BIS) was established by the WHO in 1986 and is used to determine comparative potencies of Tuberculin PPDs. Ideally, Tuberculin PPD potency should be evaluated in the species in which the tuberculin will be used but due to practical difficulties in performing potency assays in cattle, for routine PPD production, they are usually assayed in guinea pigs. Low potency tuberculin PPD is less efficient and thus inferior for bTB diagnosis. Difficulties experienced in the Irish bTB eradication programme have included the supply of sub-standard potency, and thus inferior, bovine (*M. bovis*) Tuberculin PPD in the late 1970s. The purpose of this paper is to outline the critical role of Tuberculin PPD assays carried out on naturally infected tuberculous cattle, as required by the OIE and under EU legislation in the quality control for the Irish Bovine Eradication Programme. Such assays ensure that the Tuberculin PPD used meets the diagnostic sensitivity and specificity requirements to underpin a successful national eradication programme.

## Introduction

Bovine tuberculosis (bTB) is an important infectious disease of cattle that constitutes a “One Health” concern as a public health risk due to its zoonotic potential (WHO) (1), and has significant economic and trade implications for the European Union (EU) and the World Organization for Animal Health [Office International des Epizooties (OIE)] (2, 3). Accuracy and reliability of a diagnostic test are critical in disease control and eradication strategies (2–5). Detection of the causative *Mycobacteria*, all members of the Tuberculosis complex (MTBC), during the early stages of disease is dependent on a measurement of a cell-mediated immune response *in vivo* or *in vitro*, as circulating antibodies remain undetectable until later in the disease progression (4, 6). The OIE (3) and the European Commission (2) recognize the *in vivo* intradermal tuberculin test (7) as the primary official test for the diagnosis of TB-infected animals. Annex B of the EU trade Directive (2) defines Tuberculin as “Tuberculin purified protein derivative (Tuberculin PPD, bovine or avian) is a preparation obtained from the heat-treated products of growth and lysis of *Mycobacterium bovis* or *Mycobacterium avium* (as appropriate) capable of revealing a delayed hypersensitivity in an animal sensitized to microorganisms of the same species.” The intradermal tuberculin test methodology for the diagnosis of bTB is applied in accordance with OIE guidelines in many different countries using differing applications (3). The single intradermal tuberculin test, cervical (SIT), or caudal fold (CFT), and the single intradermal comparative tuberculin test (SICTT) are widely used to detect MTBC infected animals (7) for many national programmes and for international assurance of freedom from bTB (3). Continuous evaluation of all elements, i.e., inputs, performance, and outputs, of the national disease control/eradication programme is essential to maintain effectiveness and ensure that the highest possible standards are attained and maintained (8, 9).

### Evolution of Tuberculins

Dr. Robert Koch demonstrated that *M. tuberculosis* was the causative organism of human tuberculosis (TB) in 1882. While attempting to develop a cure for TB he first produced what became known as Koch‘s old tuberculin (KOT) in 1890 from a crude extraction of heat killed cultures of *M. tuberculosis* (10–13). By 1891, KOT was being used for the diagnosis of TB in cattle and various tests applied although there were sensitivity (Se) and specificity (Sp) issues associated with it (4, 12). The first major improvement both in tuberculin production and consequent test Se and Sp was when synthetic medium was used for bacillary growth (11). Seibert introduced precipitation of tuberculo-protein in 1934 and so the term PPD was introduced (13). Tuberculin PPD had less impurities and could be standardized based on protein content. However, standardization using protein content does not necessarily correlate with the biological activity which must be routinely estimated against a reference standard (14).

The 2018 publication of Good et al. (4) detailed that in 1939 Buxton claimed that the occurrence of non-specific response could be overcome by the use of a synthetic culture medium and precipitation in the production of tuberculin and that Buxton and Glower attributed a precision of 87–97% to the tuberculin test and recommended the use of synthetic medium tuberculin. The first instructions on the performance of the SICTT issued in 1942 and detailed the conduct of the SICTT to compare the cell-mediated immune responses to separate intra-dermal injections of avian (*M. avium*) and initially mammalian (*M. tuberculosis*) and later bovine (*M. bovis*) Tuberculin PPD in each animal, to increase the specificity of the test in response to ongoing concerns over the occurrence of non-specific response in animals apparently not infected with TB (false positive) (4). In 1947, Francis confirmed that the test interpretation for the SICTT and optimal time of reading was “based on a very large number of trials followed by *postmortem* examination” (4).

In 1948, Paterson described the AN5 strain of *M. bovis* that grew as vigorously and with equivalent production capacity on synthetic medium as did *M. tuberculosis* (12). By the 1950s, bovine Tuberculin PPD, produced from *M. bovis* strain AN5, was increasingly replacing mammalian Tuberculin PPD produced from *M. tuberculosis* and was being widely used for eradication of bTB. The main advantage of bovine Tuberculin PPD was an increased Se and Sp in the diagnosis of TB in bovines over mammalian Tuberculin PPD. Hence the change to bovine PPD in the British and Irish bTB eradication programmes in the mid-1970s (15). In 1959, Paterson described tuberculin as the most important diagnostic agent in eradication schemes for bTB and it remains so today (4, 16).

Potency is a measure of a Tuberculin PPD's activity in animals sensitized with a specified organism when compared to a reference standard Tuberculin PPD (17). Ritchie pointed out that, for an effective test, it is vital to use a tuberculin of potency greater than that to which the majority of infected animals will respond (18). The use of a highly potent bovine tuberculin increases the sensitivity of the test (19) and the balance of evidence appears to favor the use of Tuberculin PPD of sufficient potency to facilitate detection of the maximum possible number of TB infected cattle for effective eradication of the disease (3, 16). Hence, the recommendations of the OIE are that national bTB eradication campaigns use doses of Bovine PPD of up to 50,000 IU/ml (20). Fears are often expressed that the use of a highly potent Tuberculin PPD will reduce the specificity of tuberculin tests and increase the false positive rate. Experience in Ireland, however, where test Sp has been demonstrated mathematically in an accepted non-disease-free population, as at least 99.95% meaning that only a fraction of 1% of the positive reactors to the SICTT are false positive and where the reliability (index of the diagnostic ability of a test) of the SICTT was determined, both in 1992 and 2011, to be in the region of 97%, would indicate that these fears are not realized (20). Experimental studies carried in Britain involving injection of tuberculous and non-tuberculous cattle with different strengths of PPDs demonstrated that the stronger the tuberculins, the better the differentiation between specific reactions (due to *M. bovis* infection) and non-specific reactions. (21). In 1993, Dr. Louis O'Reilly, the head of the TB Irish Central Veterinary research Laboratory in a report (unpublished) on an evaluation of the issue of potency and false positive results in the Irish bTB eradication programme pointed out that when Dutch tuberculin with a labeled potency of 40,000 IU/ml, which when assayed in Irish tuberculous showed 40–50,000 IU/ml was used in Ireland between May 1979 and April 1991 no problems with specificity of the test were encountered. He also commented that it was “very unlikely that the use of more potent bovine PPD will result in more false positive reactors. In fact, the numbers of false positive reactors should fall.” Use of highly potent bovine Tuberculin PPD has evidently not been an issue for Ireland, where, despite additional use of ancillary testing and more severe test interpretation reactor numbers have generally been falling since 2000 (22–24). Indeed the most recent tender for the supply of Tuberculin PPD (dated 17/05/2019) for the Irish programme specified that “Liquid Bovine PPD Tuberculin” potency “must not be <50,000” IU/ml “in tuberculous cattle (to ensure potency as assayed and used in the Eradication Programme over the last 10 years)” (25). Likewise, in GB, which uses the same Tuberculin PPD combination in the SICTT as Ireland, Goodchild et al. (26) states that in GB SICTT Sp at animal level is 99.87% (ultra-severe interpretation), and that 91.1–93.7% of reactors in GB are truly TB infected thus demonstrating that the SICTT, even using high potency bovine PPD as demonstrated by cattle assay (Table 1) retains a very high Sp with few false positive responders.

**Table 1:** Assays of bovine tuberculin PPDs in naturally TB-infected cattle: 2010–2017, PPDs used in the Irish programme; 2006, 2010, and 2018, INBS and BIS; 2004, 2008, trials involving PPDs in tuberculin testing.

**Batch no**.	**Year manufacture**	**Protein content mg/ml**	**Guinea-pig assay IU/ml**	**Bovine assay IU/ml**				
				**Year**	**95% confidence limits**			**Standard**
			Potency^*^		**Potency**	**Lower**	**Upper**	
INBS	1994	1.0	33,700	2018	30,568	19,749	46,981	BIS
BIS	1979	1.0	32,500	2013	25,821	13,792	46,561	INBS
INBS	1994	1.0	33,700	2006	46,079	31,722	68,238	BIS
170312	2017	1.0	30,350	2017	39,098	23,359	66,470	INBS
171502	2017	1.2	23,380	2017	76,927	46,043	140,170	INBS
162408	2016	0.95	24,850	2017	35,961	23,595	55,056	INBS
161102	2016	1.42	24,210	2016	50,082	30,243	86,391	INBS
153501	2015	1.25	40,950	2016	70,250	42,339	125,726	INBS
143806	2014	1.04	31,140	2015	59,058	36,584	100,423	INBS
143813	2014	1.02	30,540	2015	58,424	37,698	94,453	INBS
141310	2014	1.02	30,940	2015	52,697	34,020	84,478	INBS
141304	2014	1.01	30,640	2014	59,062	30,037	129,421	INBS
134511	2013	1.04	27,950	2014	36,822	18,300	75,367	INBS
102401	2010	1.11	26,580	2014	59,062	30,037	129,421	INBS
132203	2013	1.24	30,880	2013	52,601	35,380	80,416	INBS
132206	2013	1.08	26,900	2013	37,054	24,837	55,611	INBS
120103	2012	1.32	24,280	2012	64,795	37,264	122,280	INBS
120101	2012	1.15	21,130	2012	47,772	27,908	85,194	INBS
110404	2011	1.33	21,880	2011	50,811	30,532	88,291	INBS
110404	2011	1.33	21,880	2012	81,239	33,751	266,063	INBS
112006	2011	1.24	24,060	2012	59,999	35,112	109,705	INBS
104012	2010	1.16	23,500	2011	38,738	23,128	65,840	INBS
100308	2010	1.38	26,200	2011	57,979	34,854	102,114	INBS
102414	2010	1.11	26,220	2011	41,509	21,299	83,991	INBS
LP^a^	2008	0.16	3,400	2008	11,920	4,950	23,540	INBS
NP^a^	2008	1.23	26,380	2008	61,840	31,570	136,000	INBS
HP^a^	2008	3.11	66,700	2008	125,540	62,570	323,820	INBS
JN^b^	2004	na	10,250	2005	11,552	6,202	20,779	INBS
FM^b^	2004	na	16,500	2005	25,900	14,866	44,784	INBS
KS^b^	2004	na	9,250	2005	28,747	16,535	49,754	INBS
HT^b^	2004	na	31,500	2005	33,868	19,532	58,734	INBS
GR^b^	2004	na	27,750	2005	45,003	25,217	81,062	INBS
LP^b^	2004	na	24,500	2005	45,003	25,217	81,062	INBS

* *The Guinea Pig potency is determined against the Bovine International Standard (IS) (32,500 IU/ml)*.

a *Good et al. (19)*.

b *Good et al. (27)*.

*na, not available*.

### International Standards

To ensure uniformity in Tuberculin PPD production and use throughout the world, the WHO established International Biological Standards for potency of tuberculins. An international standard (IS) for mammalian (human) Tuberculin PPD, prepared using *M. tuberculosis*, with an assigned potency of 50,000 IU per mg, was established by the WHO in 1952 (17, 28). Similarly, an IS for avian PPD tuberculin, with an assigned potency of 50,000 I.U. per mg, was established by WHO in 1954 (29). Investigations in 1995 found the quality of the avian PPD tuberculin IS to be satisfactory both, in terms of potency and specificity per weight and the same standard is still used today (29). The Potency of a candidate Tuberculin PPD is then determined by comparing the skin reactions (after intradermal injection) to those elicited by the appropriate reference standard Tuberculin PPD of known potency in animals sensitized with a corresponding antigen. Thus, the potency of bovine Tuberculin PPD is estimated using animals sensitized to *M. bovis* and the potency of avian Tuberculin PPD is estimated in animals sensitized to *M. avium*. Potency is expressed in international units (IU) per ml; this allows comparison of tuberculins throughout the world. An international unit is a measure of the biological activity in a stated amount of the IS (17).

In 1964, the EEC adopted the Dutch National Bovine Standard, prepared from cultures of *M. bovis*, strain AN5, as the EEC standard for bovine Tuberculin PPD and assigned it a potency of 50,000 units called Community Tuberculin Units (CTU) (30, 31). An EEC working group had shown that the human Tuberculin PPD IS was not suitable for potency estimates of bovine Tuberculin PPD due to the differing dose response characteristics of both tuberculins (32).

In 1976, the WHO began an evaluation of candidate bovine Tuberculin PPDs to select a new Bovine International Standard Tuberculin PPD (BIS) (30). In 1986, a Dutch bovine Tuberculin PPD, produced in 1979 from cultures of *M. bovis*, strain AN5, was accepted as the BIS. International collaborative assays in cattle and guinea pigs against the old Dutch bovine tuberculin standard (1964) established that this new BIS had a potency of 32,500 CTU/ml and that CTU and IU for bovine Tuberculin PPD are equivalent (31).

The 2018 OIE Manual of Diagnostic Tests and Vaccines for Terrestrial Animals (20), quoting the 1968 WHO Technical Report Series No. 384 (33) states “potency testing should be performed in the animal species and under the conditions in which the tuberculins will be used in practice.” It also references the 1985 WHO Technical Report Series No. 745 (34) which provides that calibration of laboratory (in-house) “reference preparations shall be done by a number of tests” against the appropriate IS in “the animal species in which the tuberculin is to be used” and that the “control of potency of successive batches can then be carried out by biological assays in guinea-pigs, using the laboratory reference preparation.” The 2007 report of the WHO Expert Committee on the selection and use of essential medicines (35) also required that all tuberculins should comply with WHO Technical Report Series No. 745 (34). The OIE Manual (20) goes on to say “that bovine tuberculins should be assayed in naturally infected tuberculous cattle. As this requirement is difficult to accomplish, routine potency testing is conducted in guinea-pigs. However, periodic testing in tuberculous cattle is necessary and standard preparations always require calibration in cattle” (20). In addition, the routine use of cattle for potency assay purpose can be both impractical and expensive due to the lack of availability of naturally infected cattle or the costs associated with laboratory infection, thus guinea pigs are used as the alternative. Paterson recommended that guinea-pigs be used for the control at preparation/manufacture with occasional check assays in cattle but that if the type of tuberculin is changed or if a change in character is suspected that appeal must be to the assay in cattle (12).

Due to the limited supply of the BIS, the EU and OIE recommended that national and “in-house” standard Tuberculin PPDs be calibrated against the BIS and then commonly used for national and routine production potency assays. In 1994, an Irish National Bovine Standard Tuberculin PPD (INBS) was produced at CVL Lelystad and there are considerable stocks still available. Calibration against the BIS, both in guinea pigs sensitized with living *M. bovis* and naturally sensitized cattle, has shown that the INBS has a potency of 33,700 IU/ml (26). In 2018, Frankena et al. (36) suggested a new model to calibrate national and “in-house” reference standards for maximum accurately using 30 naturally TB-infected cattle (target species) and to prove the precision and accuracy of the potency estimate using 54 guinea pigs in 6 individual potency assays. The variability in potency estimates can be reduced by repeating the guinea pig assay 5 or more times on each sample (36).

### Manufacture and Composition of Tuberculins

Tuberculin PPD has been described as a poorly defined, complex mixture containing more than 100 individual components in various stages of denaturation (37, 38). Depending on where the Tuberculin PPD is to be used, or if for export to international markets, its specification must meet the relevant international standard requirements laid down by the WHO, OIE, and EU legislative requirements (2, 20, 35, 39). Tuberculins must be sterile and free from abnormal toxicity. They must also be non-antigenic, i.e., non-sensitizing when injected, so as not to cause reactions at later injections in TB-free animals. Tests for sterility, safety and sensitizing effect are set out in the OIE Manual of Diagnostic Tests and Vaccines for Terrestrial Animals 2018 (20).

### Potency

During production manufacturers are required to determine and control the potency of tuberculin batches in guinea pigs against a reference standard (34, 35). However, guinea pigs and cattle have different dose response relationships and further, there is frequently only limited agreement between the guinea pig and cattle potency assays (31, 40, 41) (Table 1). Potency estimate accuracy and agreement between calculations done in guinea pig and cattle can be improved but at the undesirable expense of conducting repeated assays using more cattle and more guinea pigs (5, 28, 36). The degree of variability in the guinea pig bioassay have been the subject of comment previously (38, 42). In recognition of this problem, Directive 64/432/EEC (2015) (2) requires the fiducial limits of error (*P* = 0.95) to be not <50% and not more than 200% of the estimated potency. The estimated potency must not be <75% and not more than 133% of the stated potency for avian tuberculin and not <66% and not more than 150%, of the stated potency for bovine tuberculin and to comply with Directive 2001/82/EC (2, 43). The tuberculo-protein content of the *M. bovis* Tuberculin PPD is adjusted based on guinea pig potency assay to achieve the target potency not <20,000 IU per ml in each final product batch. OIE recommends for bovine Tuberculin PPD that “*In cattle with diminished allergic sensitivity, a higher dose of bovine tuberculin is needed, and in national eradication campaigns, doses of up to 5,000 IU* (i.e., 50,000 IU per ml) *are recommended*” and thus bovine Tuberculin PPD with target potency exceeding 20,000 IU per ml may be sought and produced by manufacturers (20).

The method of sensitization of guinea pigs to *M. bovis* antigens can influence the results of potency assays. Repeatedly, studies have shown that the closest correlation with cattle assays is achieved by sensitization of guinea pigs with living *M. bovis* i.e., in effect infecting these guinea pigs with *M. bovis* (32, 44, 45). Sensitization of guinea pigs with heat-killed *M. bovis* or with live *M. bovis* BCG gives less reliable results, presumably because the full complement of antigens excreted during the mycobacterial multiplication stages of active infection are not produced. Likewise, potency assays performed in cattle sensitized with heat-killed *M. bovis* are not reliable (46). Cattle experimentally infected with living *M. bovis* are suitable for potency assays as are naturally infected cattle. The advantage of using naturally infected cattle from field bTB breakdowns is that this represents a more complete spectrum of exposure and stages of infection that will occur in naturally acquired infection than using a group of homogenously infected cattle.

The specificity of each production batch of bovine Tuberculin PPD is estimated in guinea pigs sensitized with heat inactivated *M. avium* according to Fishers' method (46). The skin responses elicited by the bovine PPDs are compared to those of the IS for avian PPD. The specificity of avian Tuberculin PPD is estimated in guinea pigs sensitized with living *M. bovis* using the same methodology.

Paterson, Haagsma et al., WHO, and OIE, recommended as good practice to periodically check the results of the guinea pig potency assays by estimating the potency of a proportion of production batches in naturally or artificial infected cattle (12, 20, 29–33). However, whilst this may be good practice and was provided for in the original Directive 64/432/EEC (1964) (47) and, in 1979, noted as essential by the experts in the EC sub-group of the Scientific Veterinary Commission on tuberculins (32), it was omitted when Directive 64/432/EEC was modified in 2002 (Commission Regulation 2002)^1^. The OIE Manual of Diagnostic Tests and Vaccines for Terrestrial Animals (Chapter 2.4.6—Bovine Tuberculosis) (20), still includes the recommendation to perform potency assays in tuberculous cattle, while reference is made to WHO Technical Report Series No. 745 (34).

Notwithstanding attempts by the WHO, OIE and the EU to standardize Tuberculin PPD production, quality and potency estimation, qualitative and quantitative differences between Tuberculin PPDs from different manufacturers exist. These differences occur due to various factors such as differences in manufacturing facility location, possible differences in growth media and production seed-stock strain. Differences in manufacturer's potency calibration methods, including staff experience and attention to detail, and, the quality of the reference standard used, can affect assessed potency in guinea pigs and consequential potency in cattle. In addition, the means of sensitization of guinea pigs and the number of guinea pigs used for assay by the manufacturer will affect the accuracy of the potency estimate and further result in inter-laboratory differences in potency estimates. These differences result in a wide variance both in protein content and antigenic profile and thus, differences in potency and specificity between various Tuberculin PPD products are to be expected and these, plus the relative potency of the avian and bovine Tuberculin PPDs used for SICTT and the Interferon-γ (IFN-γ) assay, will have an impact on test efficacy, Se, Sp, and Predictive Value (44, 48–52).

### The Irish bTB Eradication Programme

In Ireland, bTB is caused predominantly by infection with *M. bovis*. The Department of Agriculture, Food and the Marine (DAFM) manages the Irish bTB eradication programme which includes annual screening of all cattle herds, prompt removal of test positive animals (reactors) and animals removed for epidemiological reasons by a Veterinary Inspector or animals removed following the results of ancillary blood test(s) such as the interferon gamma (IFN-γ) assay, post-mortem surveillance by veterinary practitioners of all bovine carcases at slaughter for human consumption, movement restrictions and further consequential testing of infected herds (22–24). Good et al. in 2011 state that some 7% of cattle were positive to the single intradermal test but not to the SICTT and that various pathogenic mycobacteria e.g., *Mycobacterium paratuberculosis* subsp. *avium*, and non-pathogenic environmental Mycobacteria such as *M. hiberniae*, are abundant in the Irish environment, and cause non-specific sensitization to bovine Tuberculin PPD (20, 53). Accordingly, the SICTT is the primary screening test employed in the programme and entails ~8.5 million animal tests each year (7, 22–24). Intradermal injections of 0.1 ml of avian (25,000 IU/ml) and bovine (30,000 IU/ml) Tuberculin PPD, as assessed in guinea pigs (supplied by Prionics, Lelystad B.V.) are administered in the mid-third of the neck; the skin thickness at the site of the test is recorded at the time of injection and at test reading 72 h [±4 h] later. The nature of any reaction and the relative increase (measured in millimeters) in skin fold thickness at each injection site is evaluated at test reading. Any animal that displays clinical signs at the bovine injection site, such as oedema, exudative necrosis, heat and/or pain, in response to the injection of bovine tuberculin, at test reading is deemed test positive and therefore a “reactor” regardless of relative increase in accordance with the Directive 64/432/EEC (2015) (2). During the late 1970s, Ireland experienced problems with the low potency of tuberculin supplied for the programme. Subsequently, Ireland changed Tuberculin PPD supplier in 1980 and developed strict criteria for its requirements and incorporated potency assays on naturally infected cattle as a quality control measure (23, 24).

### Tuberculin PPD Requirements for Irish bTB Eradication Programme

Under Irish legislation, the only tuberculin that may be used in the Irish bTB eradication programme, is that supplied by DAFM. It must have marketing authorization (MA) from the Health Protection Regulatory Authority (HPRA) in Ireland in compliance with EU legislation (39, 54). Prionics Lelystad BV (previously Lelystad Biologicals BV or ID-Lelystad BV), in Lelystad, The Netherlands, has supplied the avian and bovine Tuberculin PPD used in the Irish programme since 1980 under tender.

#### Specification

The preparation, potency and labeling of Tuberculin PPD must conform to Article 51 of Directive 2001/82/EC and as specified in Directive 91/412/EEC (54). Storage must be at 4°C but, in accordance with the marketing authorization (MA), must be stable at ambient temperatures for 14 days between +2°C and +37°C.

#### Potency

Under the MA the total protein concentration of the Avian Tuberculin PPD 2500 must be between 0.5 and 0.8 mg/ml and that of the Bovine Tuberculin PPD 3000 between 1.0 and 1.4 mg/ml while at the same time:

- Bovine Tuberculin PPD supplied under MA should have a potency of 3,000 IU/dose for a 0.1 ml dose [66–150% i.e., between 1,980 IU and 4,500 IU] when tested in guinea pigs sensitized by living *M. bovis*, strain AN5 and, for the most recent tender for the supply of tuberculin PPD (dated 17/05/2019), “not be less than 50,000” IU/ml “in tuberculous cattle” and that “Potency will be determined by or on behalf of the Contracting Authority representative and prior to acceptance of any batch of tuberculin under the contract” (25) and
- Avian Tuberculin PPD should have a potency of 2,500 IU/dose for a 0.1 ml dose [75–133% i.e., between 1,875 IU and 3,325 IU] per dose when assayed in guinea pigs sensitized with heat inactivated *M. avium* and
- the pairs of Tuberculin PPDs, for the SICTT, must not exceed a maximum potency difference of 500 IU per dose (using the potency as assessed in guinea pigs) between both (Avian and Bovine Tuberculin) in the Tuberculin PPD Kit.

This stated Bovine PPD potency requirement exceeds the minimum of 20,000 IU/ml specified in Directive 64/432/EEC (2). The assays are carried out in guinea pigs as set out in EU Directive 64/432/EEC (2) using “a reference preparation of tuberculin (bovine or avian, as appropriate)” PPD “calibrated in International Units” by or on behalf of Prionics Lelystad BV.

## Cattle Potency Assay

The potency of Bovine Tuberculin PPD 3000 supplied for use in Ireland is estimated in Lelystad in guinea pigs sensitized with living *M. bovis* relative to the BIS PPD on behalf of the manufacturer. The potency of one or more supplied batches of Tuberculin PPD is checked in Ireland in naturally infected cattle each year against the INBS, which was calibrated in 1994 in cattle and guinea pigs (28). The potency of Tuberculin PPDs used in various trials involving tuberculin testing in Ireland has also been assayed (19, 27). The INBS tuberculin is also periodically assayed against the BIS in naturally infected Irish cattle in Ireland and in guinea pigs by Prionics Lelystad BV in accordance with EU Directive 64/432 (2). A recent assay included the INBS against the BIS in 2018 in naturally infected Irish cattle indicated a potency of 32,265, an earlier assay in 2006 had shown a potency of 46,079 and an assay of the BIS against the INBS in 2013 had shown a potency of 25,821 (Table 1). The results of these latter two assays caused some concern. Subsequently, at the *M. bovis* conference in Cardiff in 2014 (55) problems were reported where evident visual differences and even non-visual deterioration of the BIS in some ampoules supplied by the NIBSC was resulting in highly variable potency assay results being obtained in guinea pigs, when injecting identical amounts from different vials. Dr. Bakker reported that in the deteriorating vials the BIS was no longer completely water soluble and contained varying amounts of large particles which affected the potency assay and while these could be centrifuged (3,000×g) and removed there was then a loss of antigen (55). Consequently, the NIBSC removed the visibly deteriorated ampoules from their stocks and, the OIE established in 2015 an *ad hoc* working group with the task of finding, a new source of a bovine PPD and work is currently underway to develop a new BIS.

In Ireland cattle for assays are chosen in accordance with the Standard Operating Procedures for Tuberculin Potency Assays on TB cattle in the isolation unit at the DAFM Research Farm the purpose of which are to ensure the welfare of the cattle and the integrity of the assays carried out. Cattle assays are subject to strict individual project licensing conditions which are issued and audited by the HPRA.

Cattle, from TB-infected farms of origin, which have given a positive result to a SICTT, which have a skin-fold thickness measurement increase at the bovine injection site, which is more than 4 mm greater than the increase at the avian injection site and are positive to IFN -γ BOVIGAM^®^ (Prionics, Lelystad B.V.) assay (56), are selected for assays at the isolation unit. For ease of handling and husbandry, young steers from ~6-months to 2-years of age are usually selected. The interval between the SICTT on the farm of origin and the potency assay must be at least 60 days (57). The animals are normally kept for a maximum of two assays or for up to a year and replaced as necessary by further field test positives as above.

Potency assays on batches of routine issue bovine Tuberculin PPD are carried out under license 2–3 times each year depending on availability of sufficient numbers of suitable field reactor cattle. At each assay, the potencies of three selected test batches are estimated against the IBNS bovine Tuberculin PPD with an assayed potency of 33,700 IU/ml. Each ampoule of the freeze-dried IBNS contains 1.8 mg PPD in a glucose phosphate buffer containing phenol (28). Dilution to 1 mg/ml is prepared, by adding 1.8 ml of distilled water. Isotonic phosphate-buffered saline, pH 7.3 is used to prepare the 20% dilutions of all the Tuberculin PPDs for the assay representing 0.2 mg/ml protein concentration (30). Each of the 3 Tuberculin PPDs for assay and the comparator INBS and/or BIS, as relevant, is used at two dilutions corresponding to protein concentrations of 1.0 and 0.2 mg/ml.

**Figure 1:**
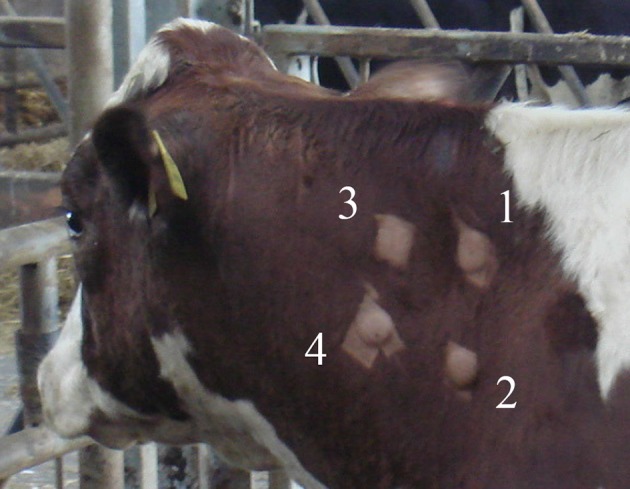
Injection sites with reactions, showing injection/measurement sequence,1–4 (on each side of the neck), for bovine tuberculin PPD assay.

Thus, there are eight tuberculin preparations (4 undiluted and 4 diluted) which are inoculated into each animal at four sites on each side of the neck as shown in Figure 1. The distance between the injection sites is ~10–12 cm.

A group of eight cattle is required to rotate all 8 injections (i.e., the reference standard and 3 test PPDs each at two dilutions) sequentially through each of the eight possible neck sites using the Latin square design. This is necessary to take account of the different sensitivity at different sites on the bovine neck (12, 17). The Tuberculin PPDs are allocated to sites based on a randomized schedule laid out in advance in the assay worksheets. For routine assays, three groups of eight cattle are used to increase the reliability of the assay to acceptable levels of accuracy i.e., for the same reasons as recommended by Frankena et al. for the guinea pig assay (36). A separate worksheet, each with a unique pattern of allocation of Tuberculin PPDs to neck sites is used for each group.

The injection sites are clipped and the skin-fold thickness at each injection site is measured using a caliper with 1 mm graduations at 0 h. Using McLintock syringes, 0.1 ml of each Tuberculin PPD are injected intradermally. All aspects of each test (tuberculin administration, initial and subsequent skin measurement) on each assay animal are conducted by the same veterinarian. Each injection site skin thickness measurement is taken and recorded at 0 and 72 h. Table 2 shows an example of the skin measurement data recorded for one of the 3 groups of 8 cattle on a recent assay.

**Table 2:** Example of injection site measurement data for a group of 8 bovines in a potency assay on bovine tuberculin PPDs.

**Group 2**	**Irish standard**						**Test PPD L**						**Test PPD M**						**Test PPD N**					
	**1.0 mg**			**0.2 mg**			**1.0 mg**			**0.2 mg**			**1.0 mg**			**0.2 mg**			**1.0 mg**			**0.2 mg**		
**Animal ID**	**Hour**		^*^Inc	**Hour**		^*^Inc	**Hour**		^*^Inc	**Hour**		^*^Inc	**Hour**		^*^Inc	**Hour**		^*^Inc	**Hour**		^*^Inc	**Hour**		^*^Inc
	**0**	**72**		**0**	**72**		**0**	**72**		**0**	**72**		**0**	**72**		**0**	**72**		**0**	**72**		**0**	**72**	
1038	12.5	19	6.5	13	18	5	13.5	18	45	11	17	6	10	16.5	6.5	12	24	12	11	18	7	13	16	3
2190	11	16	5	9	12	3	10.5	14	3.5	9	12.5	3.5	10	15	5	10	14	4	9	12	3	11	16	5
1629	7	13	6	10	15	5	8	14	6	10	14	4	10.5	16	5.5	8	14	6	10	14.5	4.5	6	10	4
1664	8	16	8	8.5	16.5	8	7	13	6	7.5	15.5	8	9	19	10	7	13	6	9	19	10	8	15	7
1636	8	12.5	4.5	6	11	5	8	12	4	7.5	12	4.5	7	14	7	8	10.5	2.5	6.5	12	5.5	9	11	2
1658	9	16	7	9	15.5	6.5	8	16	8	11	18.5	7.5	9	20	11	8	13	5	12	19	7	9	13	4
2256	9	18	9	11	18.5	7.5	10	20	10	10	16	6	9	19	10	11	17	6	10	17.5	7.5	10	14.5	4.5
2175	7	12.5	5.5	9	12	3	9	16	7	9	15	6	8	14	6	9	13.5	4.5	9	18	9	7.5	12	4.5

* *Increase in skin fold measurement millimeters at 72 h (72 h.mms−0 h.mms = Inc.mms)*.

Measurements, recording increases at the various injection sites for each dilution of PPD being assayed and the reference standard, are analyzed using standard statistical methods for parallel-line assays (58), using the GLM procedure in SAS v9.1 (59). Site of injection and side of the neck are included in the final model if significant (*P* < 0.05). The 95% confidence limits for the relative potency are calculated according to Fishers' method (46). This analysis estimates the potency of the three routine issue Tuberculin PPDs as compared to the IBNS Tuberculin PPD (46). Potency is expressed in IU/ml, based on the potency of the INBS at 1.0 mg/ml of 33,700 IU/ml as calibrated previously against the BIS (28). Table 3 shows the results of the analysis for the assay referred to on Table 2.

**Table 3:** Example of relative potency and calculated international units of the 3 test bovine tuberculin PPDs in Table 2.

	**Relative potency (%)**			**International units (IU/ml)**		
		**95% confidence limits**			**95% confidence limits**	
**Test PPD**	**Mean**	**Lower**	**Upper**	**Mean**	**Lower**	**Upper**
L	109.3	543	223.6	36,822	18,300	75,367
M	175.3	89.1	384	59,062	30,037	129,421
N	118.2	59.1	244.2	39,839	19,921	82,288

Guinea pigs and cattle have different dose response relationships and unless multiple groups of cattle and guinea pigs are used both accuracy of the potency estimate and the potency correlation between the species is poor (11, 28, 29, 32, 36, 54, 60). Notwithstanding multiple assays in each species, the potency estimated in the guinea pig (five assays each with 9 guinea pigs), differ from those obtained in cattle assays (three assays each with 8 cattle) as evidenced by the results shown in Table 1. At least some of the difference is likely to be due to the BIS being used as the reference standard for the guinea pig assay. The BIS, as stated previously has been deteriorating from prior to 2005 when it was first publicly reported (36), and has since been giving highly variable potency assay results in guinea pigs, when injecting identical amounts from different vials (55). However, successive assays have shown (Table 1) that batches that meet the required potency of 30,000 [66–150%] IU/ml in guinea pig assays also attained or exceeded this potency when assayed in cattle and indeed in recent years frequently attains or exceeds the potency recommended by the OIE for use in bTB Eradication programmes. From the results presented in Table 1, it would appear likely that the assay results in guinea pigs are underestimating the potency of the assayed PPDs by varying amounts most likely depending on the degree of deterioration of the BIS in the vial used for the assay.

## Discussion

As early as 1908, it was lamented that “some of the tuberculin on the market is impotent and worthless” and Buxton also commented on tuberculin quality in 1934 (60–63). In 2011, Good (19) compared “the impact of different potencies of a single bovine PPD tuberculin on the field performance of the” SICTT and SIT and found “a significant difference in the number of reactors detected using the high and low potency tuberculins.” This study also found that the low potency tuberculin, although not the lowest detected commercially available, missed detecting animals which, having negative responses at the bovine injection site, would individually have qualified for OIE certification as bTB-free for export purposes, despite subsequently being found to have multiple tuberculous lesions visible at routine slaughter (3, 5, 19).

When the United Kingdom (UK) switched from using Weybridge Tuberculin PPD to Dutch Tuberculin PPD, it was found that the tuberculin manufacturing source influenced both the Se and Sp of the SICTT (63). Data from international studies indicate a sensitivity range, at individual animal level, of 68–96.8% and 96–98.8% specificity for the CFT (80–91% sensitivity and 75.5–96.8% specificity), for SIT and, for the SICTT (55.1–93.5% sensitivity and 88.8–100% specificity) (7, 60, 64). The caudal fold has been repeatedly determined to be the least sensitive site available for intradermal test and hence, the CFT requires higher potency tuberculin to achieve an acceptable Se for use in bTB control/eradication programmes; the mid third of the neck proved to be the most sensitive site for the intradermal test (4). Differences in test sensitivity and specificity are largely due to bTB prevalence, variation in testing techniques, differences in tuberculin doses, Tuberculin PPDs with differing antigenic profiles, Tuberculin PPD potency, relative potency of avian, and bovine PPDs in the comparative test, the interpretation of skin reactions, and the prevalence of non-specific or cross-reactive antigens in the environment (19, 23, 38, 50). Different findings of various studies are attributed to multiple factors including differences in study design, the selection of animals in various stages of infection, frequency of testing and most significantly, how the infection status of the animals was determined. The specificity of the test will be affected by sensitization to environmental mycobacteria or other organisms that have shared antigens with *M. bovis* (65). The level of cross-sensitization will vary from region to region. In Ireland, exposure of cattle to multiple environmental mycobacteria may result in cross-reactions to bovine PPD (53), nevertheless, as previously stated, the reliability of the SICTT, being a relatively crude index of the diagnostic ability of a test based on the Se and Sp of the test in the environment in which it is used, has been assessed as being in the region of 97% (20, 66).

Few, if any, studies discussing the sensitivity range, and comparing *ante-mortem* test outcomes in various countries or regions or over time consider differences in the manufacturer or potency of the tuberculin as critical to test Se and much of the more recent literature in particular, seems to assume that all tuberculins will perform equally whether in skin test or IFN-γ assay (48, 67–71). For example, the 2012 EFSA scientific opinion states that the selection of the cattle populations, the bovine TB testing history of the cattle and the prevalence of environmental mycobacteria, may have influenced performance estimates in the surveillance population samples used in their latent class analysis, specifically mentioning the low sensitivity of the skin test in one dataset (68). However, they fail to mention as pertinent that the tests they compared in the datasets used tuberculin, avian and bovine PPDs, from 3 different manufacturers and with, at least the bovine Tuberculin PPD, at 2 different stated potencies, with, at the time the data was generated, a different manufacturer's Tuberculin PPDs used in each jurisdiction. In the context of tuberculin used in the IFN-γ assay for example, Tameni et al. (51) commented that wide fluctuations of the results of the IFN-γ assay had been traced back to the use of different PPD batches and that tuberculins were not prone to easy standardization of their antigenic content. Casal et al. (69) compared the performance of the SIT, ID-Vet IFN γ, and the Bovigam^®^ noting that “Over the 113 cattle with confirmed bTB (group 2), 32 (28.3%) were classified as positive reactors by Bovigam^®^ but negative to the SIT test;” and in the same group “36 cattle (31.9%) were positive with Bovigam^®^ (0.05 cut-off point) but negative to IDvet IFN-γ assay (35% s/p cut-off point).” However, similar results were achieved between the IFN-γ assays applying the 0.1 cut-off point in the Bovigam and the S/P ratio of 16 in the IDvet test. These results were comparable to the results obtained by de la Cruz et al. (70) who also found the IDvet IFN-γ assay less sensitive than the Bovigam^®^ but that the Se of the IDvet IFN-γ assay might be improved by adjusting the cut off points. The Bovigam TB kit flier states that BOVIGAM Tuberculin PPD produced by Prionics Lelystad B.V. uses bovine Tuberculin PPD at a potency of 30,000 IU/mL and avian Tuberculin PPD at a potency of 25,000 IU/mL (https://assets.thermofisher.com/TFS-Assets/LSG/Flyers/animalhealth_flier_bovigam_tb_CO121138.pdf). The IDvet brochure states that their IDvet test for detecting the cellular response to *Mycobacterium bovis* uses bovine PPD as the specific antigen source and avian PPD as the non-specific antigens source with matched potencies of bovine and avian tuberculins but the potency/ml is not stated (https://www.id-vet.com/wp-content/uploads/2014/07/brochure_IFNG_BovineTB_doc250.pdf). When discussing the Se, Sp and efficacy for the detection of TB infected animals of the various tests, neither set of authors considered the source of the Tuberculin PPDs, avian, and bovine, the individual potency or the relative potencies of these PPDs, in the possible reasons for the differences in observed results of the test performances. Similarly, Keck et al. (71) observed very low SIT positive rates during two screening campaigns where the use of the Bovigam^®^ assay was found to increase the sensitivity of TB detection by more than 30% over and above the SIT using the official bovine Tuberculin PPD and the effect of the different PPDs used was not discussed as a possible factor in the different Se observed (71).

Largely, due to the ill-defined nature of the antigens in Tuberculin PPD as well as the complexity of Tuberculin PPD production, to date, there has been little progress in improving Tuberculin PPDs to enhance test specificity and sensitivity (37, 52). Successful eradication of bTB has been achieved in many countries by the rigorous application of tuberculin testing and the culling of reactor cattle. While the quality of the Tuberculin PPD used is undoubtedly critical for test efficacy for bTB control and eradication programmes and to underpin certification of disease freedom at animal and herd level, comparisons of commercially available tuberculins, has shown the potency of bovine tuberculins and, to a lesser extent, avian tuberculins varied widely such that the majority would not have met the required minimum dose of 2,000 IU if applied as the standard 0.1 ml dose (5, 42). The use of tuberculins with inferior potency has direct implications for the diagnosis of bTB and for the surety of consequent certification of herd and animal disease freedom (5, 19). While the European Pharmacopeia, WHO, OIE, and EU have established the standard for tuberculins (2, 20, 33–35, 39, 43, 54), there is no independent body evaluating commercially available preparations or establishing and maintaining standards of Tuberculin PPD potency akin to The International Organization for Standardization (https://www.iso.org) ISO which is designated to independently assess and attest the standards claimed by the manufacturers. There may be a potential role for the European and/or OIE Tuberculosis Reference Laboratories in the verification of tuberculin potency. It would reasonably be expected that when standards are not complied with that the authorities should take steps to ensure that such products are precluded from use. It would also undoubtedly be desirable to have an alternative methodology for PPD potency assay less dependent on infecting Guinea Pigs and the availability of TB infected cattle. Due to potency issues with tuberculin supply in the past and considering the above publications demonstrating that potency is critically important in test efficacy, Ireland has, using naturally infected tuberculous cattle, maintained an independent check of the potency of the bovine Tuberculin PPD supplied under the Irish programme.

## Data Availability Statement

All datasets generated for this study are included in the manuscript/supplementary files.

## Ethics Statement

The Tuberculin PPD assays and use of animals therein was reviewed by Department of Agriculture and Marine/University College Dublin ethics committee as part of the Health Products Regulatory Authority (HPRA) project authorization application process. The HPRA is the competent authority in Ireland responsible for the implementation of EU legislation (Directive 2010/63/EU) for the protection of animals used for scientific purposes. HPRA are committed to ensuring that the care and use of animals for scientific purposes is in line with the 3R principles—Replacement, Reduction, and Refinement. Accordingly, projects and individuals require authorization to carry out research using animals including authorization of the establishment where the animals are kept. The use of animals for Tuberculin PPD potency assay, the persons who perform the assays and the premises where the animals are kept, are authorized (licensed) by the HPRA and subject to strict individual project licensing conditions which include reporting and annual audit.

## Author Contributions

AD conceived the study. AD, DB, and MG analyzed the data. AD and MG carried out a literature search and wrote the initial manuscript. All the authors participated in reviewing, editing, read and approved the final draft, and collaborated in producing the final version.

### Conflict of Interest

The authors declare that the research was conducted in the absence of any commercial or financial relationships that could be construed as a potential conflict of interest.

## Footnotes

^1^Commission Regulation (EC) No 1226/2002 of 8 July 2002 amending Annex B to Council Directive 64/432/EEC OJ L 179, 9.7.2002, p. 13–18.
